# La fibrillation atriale, fréquence, facteurs étiologiques, évolution et traitement dans un service de cardiologie de Dakar, Sénégal

**Published:** 2010-08-25

**Authors:** Alassane Mbaye, Soulemane Pessinaba, Malick Bodian, Bamba Ndiaye Mouhamadou, Fatou Mbaye, Adama Kane, Valentin Yaméogo Nobila, Maboury Diao, Abdoul Kane

**Affiliations:** 1 Faculté de Médecine, Pharmacie et Odontologie, Université Cheikh Anta Diop. Service de cardiologie, Hôpital Général de Grand Yoff, Dakar (Pr Abdoul Kane) BP: 3270 Dakar-Sénégal

**Keywords:** Arythmie cardiaque, fibrillation atriale, valvulopathie rhumatismale, Sénégal

## Abstract

**Introduction:**

La fibrillation atriale est le trouble du rythme cardiaque soutenu le plus fréquent. Les objectifs de ce travail étaient d’étudier la fréquence, les facteurs étiologiques, l'évolution et le traitement de la fibrillation atriale dans un service de cardiologie de Dakar au Sénégal.

**Méthodes:**

Il s'agit d'une étude rétrospective réalisée sur cinq ans et portant sur l’analyse de 150 dossiers de patients hospitalisés pour une fibrillation atriale.

**Résultats:**

La fréquence de la fibrillation atriale était de 5,35 % et représentait 66% des troubles du rythme cardiaque avec une prédominance féminine (sex-ratio H/F = 0,45) et un âge moyen de 57,06 ± 18,64 ans. L’origine de la fibrillation était valvulaire dans 36,7% des cas. Les complications thromboemboliques étaient plus fréquentes dans la fibrillation atriale non valvulaire (p=0,006), chez les sujets d'âge > 50 ans (p=0,01) et en cas de dilatation de l’oreillette gauche (p=0,05). Une anticoagulation par anti-vitamine K était instaurée dans 62% des cas et un traitement de contrôle de la fréquence cardiaque dans 87,33%.

**Conclusion:**

La fibrillation atriale est fréquente en milieu hospitalier africain et concerne des sujets relativement jeunes. L’hypertension artérielle et les valvulopathies rhumatismales sont les principaux facteurs étiologiques. Le traitement reste essentiellement médicamenteux par contrôle de la fréquence cardiaque.

## Introduction

La fibrillation atriale (FA) est une arythmie supra-ventriculaire caractérisée par une activité électrique atriale anarchique et chaotique avec pour principale conséquence une altération de la fonction mécanique des oreillettes [1]. Les complications thrombo-emboliques, notamment cérébrales et les conséquences hémodynamiques font la gravité de la FA. Conséquence du développement de la maladie coronaire en Afrique [2] et de la persistance des valvulopathies rhumatismales, la FA est de plus décrite chez le Noir Africain [3,4]. Les objectifs de ce travail étaient d’étudier la fréquence, les facteurs étiologiques, l'évolution et traitement de la FA dans un service de cardiologie d'un hôpital universitaire du Sénégal.

## Méthodes

Il s’agit d’une étude rétrospective descriptive réalisée entre janvier 2003 et décembre 2007. Elle porte sur 150 dossiers de patients admis pour une FA confirmée à l’électrocardiogramme (ECG) au service de cardiologie de l’Hôpital Général de Grand Yoff à Dakar au Sénégal. Nous avons analysé les aspects cliniques, démographiques, électrocardiographiques, échocardiographiques, évolutifs et thérapeutiques. Ces données ont été analysées à l’aide du logiciel SPSS 15.0 For Windows. Le test de chi-carré (S2) était utilisé pour la comparaison des moyennes avec un seuil de significativité pour une valeur de p inférieure à 5% (p < 0,05).

## Résultats

**Les caractéristiques générales de la population étudiée**

Un total de 150 patients dont 103 femmes étaient hospitalisés pour une FA dont la fréquence était de 5,35% correspondant à 66% des patients hospitalisés pour un trouble du rythme. L'âge moyen était de 57,06 ± 18,64 ans (17-91 ans). Les antécédents étaient dominés par l’hypertension artérielle et les valvulopathies (Tableau 1).

La FA était symptomatique dans 86,7% des cas alors qu'elle était de découverte fortuite dans 15,3%. La fréquence cardiaque moyenne auscultatoire était de 100,85 ± 24,40 battements/min. La fréquence ventriculaire moyenne à l'ECG était de 107,37 ± 29,80 cycles/min. Le Tableau 2 résume les résultats de l’échocardiographie. La fraction d'éjection ventriculaire gauche était étudiée par la méthode de Teicholz et la dysfonction ventriculaire gauche était plus fréquente chez les patients présentant une FA non valvulaire (p=0,047). La taille de l'OG était appréciée par la mesure de son diamètre antéro-postérieur systolique en coupe para-sternale grand axe et en mode TM. On notait une dilatation de l'OG plus fréquente dans la FA valvulaire (p = 0,004). L’échocardiographie trans-œsophagienne réalisée dans 26 cas, montrait un contraste spontané dans 17 cas et un thrombus constitué dans 2 cas.

**Facteurs étiologiques de la FA**

La FA était d’origine valvulaire rhumatismale dans 36,7% des cas et non valvulaire dans 63,3% avec 6,7% de FA idiopathique. La valvulopathie mitrale était l'atteinte valvulaire la plus fréquente et l’hypertension artérielle la cause non valvulaire la plus fréquente (Tableau 3).

**Le traitement**

Tous les patients ont bénéficié de traitement par héparine de bas poids moléculaire en hospitalisation avec un relais par une anticoagulation par anti-vitamine K (AVK), en prévention des accidents thrombo-emboliques chez 62 % d'entre eux. Un traitement de contrôle de la fréquence cardiaque était prescrit dans 131 cas (87,33%). Une cardioversion pharmacologique par Amiodarone per os (15-30mg/Kg en dose de charge puis 600mg/jour) était réalisée chez 10 patients dont 6 avec succès et une cardioversion électrique réussie dans 2 cas.

**évolution hospitalière**

La durée moyenne d’hospitalisation était de 14,36 ± 6,90 jours. La FA était réduite dans 12 cas dont 4 cas spontanément (2,7%) et 8 cas (5,3%) après cardioversion. Ainsi la FA était paroxystique dans 2,7% des cas, persistante dans 5,3% et chronique dans 92% (138 cas). Parmi les complications on notait 17,33% d’insuffisance cardiaque, 14,7% d’embolie cérébrale (22 cas) et 16% de thrombose intracardiaque. L'embolie cérébrale était plus fréquente dans les cas de FA non valvulaire (p=0,006), chez les sujets d'âge > 50 ans (p=0,01) et chez ceux ayant une dilatation de l’OG (p=0,05).

## Discussion

La FA est le trouble rythmique cardiaque soutenu le plus fréquent, en particulier chez les sujets âgés ou porteurs de cardiopathie. Son épidémiologie et ses facteurs étiologiques sont variables d’un pays à un autre. Les données épidémiologiques sur la FA concernent souvent des sujets caucasiens américains ou européens où plus du tiers des consultations motivées par une arythmie cardiaque est causé par une FA [1,5] avec une prévalence estimée à 1% dans la population générale et une augmentation avec l'âge [1]. L'âge moyen étant de 75 ans [1,6], nettement supérieur à celui retrouvée dans notre série. En Afrique subsaharienne, les données existantes concernent des séries hospitalières avec une prévalence de l'ordre de 7% avec une population relativement jeune [3,4] comme le rapportent certaines séries asiatiques [7, 8]. Bien que ne reflétant pas la prévalence exacte de la FA dans la population générale, ces données hospitalières donnent au moins un aperçu sur la réalité de ce trouble du rythme cardiaque chez le Noir qui serait décrit comme étant moins exposé au risque de FA [1]. Les facteurs étiologiques de la FA sont nombreux et variables [9-114]. Dans l’étude Framingham [12], l’hypertension artérielle, la décompensation cardiaque et les valvulopathies étaient les principales causes d’apparition de la fibrillation atriale. Les facteurs associés à la FA non valvulaire largement étudiés sont dominés par l’hypertension artérielle, l’âge avancé, l’infarctus du myocarde, l’insuffisance cardiaque, l’hypertrophie ventriculaire gauche, la dilatation de l’oreillette gauche (OG), le diabète et l’hyperthyroïdie [13-17].

En Afrique, malgré la progression de la maladie coronaire, les valvulopathies rhumatismales restent encore une cause non négligeable de FA, de l'ordre de 25% dans deux séries africaines [3,4]. Mais quel qu'en soit l'origine, la FA est associée, à long terme, aux risques de complications notamment cardiaques et cérébrales. Le risque thrombo-embolique dépend directement de la présence de facteurs pouvant entrainer un accident vasculaire cérébral sévère avec un risque élevé de mortalité précoce et d'handicap moteur [12,16,18]. Dans l’étude de Framingham [12], le risque d’accident ischémique est multiplié par 5,6 en cas de FA non rhumatismale et par 17,5 dans la FA d’origine rhumatismale. Chez nos malades, les facteurs positivement associés à l'accident vasculaire cérébral étaient la valvulopathie rhumatismale (p=0,006), l’âgé > 50 ans (p=0,01) et la dilatation de l’OG (p=0,05). La prévention de ces complications repose sur l'anticoagulation au long cours par les médicaments anti-vitamine K (AVK) et plus récemment les nouveaux anticoagulants oraux ou sur les antiagrégants plaquettaires en alternative aux AVK [19-21]. Ce traitement constitue un enjeu important car permettant de réduire la létalité liée à la FA et de lutter contre l’accident vasculaire cérébral, première cause de dépendance chez le sujet âgé [21].

Un autre problème qui se pose dans le traitement médicamenteux de la FA est celui du choix entre le contrôle de la fréquence ventriculaire et le maintien en rythme sinusal, cela en termes d’efficacité, de sécurité et de qualité de vie pour le patient [22-24]. Le ralentissement et le contrôle de la réponse ventriculaire par un traitement médicamenteux sont aussi efficaces que la cardioversion pour la restauration du rythme sinusal [22,23]. Dans notre série comme dans d'autres séries africaines [3,4], le choix thérapeutique était essentiellement porté sur le contrôle de la fréquence. Ce traitement ralentisseur de la fréquence cardiaque peut être proposé en première intention aux patients peu ou pas symptomatiques et ayant un risque élevé de récidive [22]. Des traitements non pharmacologiques (ablation et chirurgie) se sont développés et sont une alternative aux échecs du traitement médicamenteux [25,26]. Toutefois la bonne prise en charge des facteurs de survenue de la FA reste un enjeu capital et pourrait ouvrir la voie à de nouvelles perspectives de prévention pharmacologique de la FA [27].

## Conclusion

La persistance de la maladie rhumatismale et le développement des coronaropathies en Afrique font de la fibrillation atriale un trouble du rythme cardiaque fréquent en milieu hospitalier. Elle touche des sujets relativement jeunes et les facteurs étiologiques sont dominés par les valvulopathies rhumatismales et l’hypertension artérielle. Le risque d'accidents vasculaires cérébraux doit justifier une prise en charge efficace pour la prévention de ces complications qui sont potentiellement graves et invalidantes.

## Conflits d'intérêts

Les auteurs ne déclarent aucun conflit d’intérêts.

## Contribution des auteurs

Tous les auteurs ont contribué à la ce travail et à la rédaction du manuscrit selon les critères définis par l’ICMJE.

## Figures and Tables

**Tableau 1:** Antécédents médicaux des patients en Fibrillation atriale (n=150)

**Antécédents**	**Nombre**	**Pourcentage (%)**
Hypertension artérielle	61	40,7
Valvulopathie	55	36,7
Cardiomyopathie dilatée	7	4,7
Cardiopathie ischémique	2	2,7
Hyperthyroídie	7	4,7
Cœur pulmonaire chronique	3	2
Embolie pulmonaire	2	1,3
Asthme	2	1,3
Péricardite	1	0,7
Wolff Parkinson White	1	0,7
Bloc auriculo-ventriculaire complet	1	0,7

**Tableau 2:** Paramètres échocardiographiques mesurés par voie trans-thoracique

**Paramètres échocardiographiques**	**moyenne**	**Écart-type**
Diamètre antéro-postérieur de l'OG (mm)	49,84	10,76
Diamètre télé-diastolique du VG (mm)	51,58	9,89
Diamètre télé-systolique du VG (mm)	35,63	10,03
Fraction de raccourcissement VG (%)	32,4	9,75
Fraction d'éjection du VG (%)	56,9	13,7
Diamètre moyen du ventricule droit (mm)	26,65	7,87
PAPS (mm Hg)	53,53	23,95

OG: oreillette gauche; VG: ventricule gauche; PAPS: pression artérielle pulmonaire systolique

**Tableau 3:** Facteurs étiologiques de la FA selon les tranches d'âge (N = 150)

**Facteurs étiologiques**	**17-39 ans (n=30)**	**40-59 ans (n=44)**	**≥ 60 ans (n=76)**
**FA valvulaire**			
Valvulopathie mitrale	24 (80%)	24 (54,54%)	
Valvulopathie aortique	7 (23,33%)	11 (25%)	
**FA non valvulaire**	Hypertension artérielle	6 (13,63%)	55 (72,36%)
Cardiomyopathie ischémique		1 (2,3%)	5 (6,58%)
Cardiomyopathie dilatée	2 (6,7%)	5 (11,36%)	
Péricardite aigue	3 (10%)	3 (6,81%)	1 (1,31%)
Cœur pulmonaire chronique	1 (3,33%)	1 (2,3%)	5 (6,58%
Hyperthyroídie	2 (6,7%)	3 (6,81%)	2 (2,62%)
Diabète		1 (2,3%)	3 (3,93%)
Pré-excitation ventriculaire	1 (3,33%)		
FA idiopathique		4 (9,09%)	6 (7,9%)

FA: fibrillation atriale, NB: les valeurs sont exprimées en nombre et en pourcentage (parenthèses) de l'effectif par tranche d'âge
